# Transcriptomic profile of microglia following inflammation-sensitized hypoxic-ischemic brain injury in neonatal rats suggests strong contribution to neutrophil chemotaxis and activation

**DOI:** 10.1186/s12974-025-03516-1

**Published:** 2025-07-19

**Authors:** Anna-Sophie Bremer, Nico Henschel, Hannah Burkard, Maria Eugenia Bernis, Thomas Ulas, Hemmen Sabir

**Affiliations:** 1 Department of Neonatology and Pediatric Intensive Care, Children’s Hospital, University of Bonn, 53127 Bonn, Germany; 2 German Center for Neurodegenerative Disease (DZNE), 53127 Bonn, Germany; 3 Systems Medicine, German Center for Neurodegenerative Diseases (DZNE), 53127 Bonn, Germany; 4 PRECISE Platform for Single Cell Genomics and Epigenomics, German Center for Neurodegenerative Diseases, West German Genome Center, the University of Bonn, Bonn, Germany; 5 Genomics and Immunoregulation, Life & Medical Sciences (LIMES) Institute, University of Bonn, 53115 Bonn, Germany

**Keywords:** Neonatal Hypoxia-Ischemia, Brain inflammation, Microglia priming, Neutrophil recruitment, Transcriptomic analysis

## Abstract

**Background:**

Inflammation-sensitized hypoxic-ischemic brain injury significantly contributes to neonatal mortality as affected neonates do not benefit from standard cooling treatments. To get further insight into inflammatory responses involved, we experimentally investigated the immune response of microglia in an inflammation-sensitized neonatal hypoxia-ischemia (HI) model.

**Results:**

Transcriptomic analysis of microglia isolated from brains following inflammation-sensitized HI brain injury revealed a strong upregulation of leukocyte recruitment and pro-inflammatory markers. Specifically, markers associated with neutrophil-mediated immune responses and chemotaxis were upregulated in the inflammation-sensitized HI group compared to the non-inflammation-sensitized HI and control groups. Serpine 1 and Selp could be identified as specifically upregulated markers indicating an acute inflammatory condition before HI injury.

**Conclusion:**

Our study revealed preliminary data about a microglia population which is primed to recruit peripheral neutrophils to infiltrate the brain and mediate neutrophil immune response. We showed a contribution to neutrophil activation in case of inflammation following HI in the brain. Targeting microglia-mediated neutrophil recruitment can indicate a possible treatment approach in case of inflammation-sensitized HI brain injury.

**Supplementary Information:**

The online version contains supplementary material available at 10.1186/s12974-025-03516-1.

## Background

Neonatal hypoxic-ischemic (HI) encephalopathy remains one of the leading causes of morbidity and mortality in infants worldwide, particularly in countries where intensive care is not generally available. The standard treatment, therapeutic hypothermia, is beneficial in only approximately 30% of cases and is known to be ineffective in presence of perinatal infections. Currently, no treatment exists for infants simultaneously suffering from both perinatal infections and HI encephalopathy. Therefore, there is an urgent need to identify early regulatory biomarkers and treatment strategies for this specific condition. A deeper understanding of the inflammatory responses in the brain is essential to comprehend the unique challenges caused by perinatal infections and HI injury.

## Introduction

Neonatal hypoxia-ischemia (HI) is one of the leading causes of neonatal encephalopathy and can lead to neonatal mortality and livelong morbidities [1]. The only standard treatment available to treat newborns suffering from moderate HI is therapeutic hypothermia (TH) with a therapeutic time window of 6 h [2]. TH is beneficial in only approximately 30% of the cases and even less beneficial in low- and middle-income countries where perinatal infections are significantly higher [3]. Studies in newborn rats have shown that TH is ineffective in the presence of inflammation [4, 5]. To identify specific regulators or biomarkers to indicate which neonate might benefit from TH, better insight into the pathological mechanism underlying HI in the presence of infection is necessary. Therefore, a neonatal animal model of inflammation-sensitized hypoxic-ischemic brain injury, in which TH was not neuroprotective, was established in newborn rats [4, 6].

In the field of neuroinflammation, microglia are the most studied resident immune cells because they play a key role in the immune response of the brain. As microglia are highly heterogeneous and can be divided into different subpopulations based on their marker expression [7, 8], they can strongly support both pro- and anti-inflammatory pathways, contributing to versatile disease outcomes [9–11].

Previous studies have shown early activation of microglia and a significant increase in pro-inflammatory cytokines in the brains of inflammation-sensitized neonatal rats 24 h post HI [4, 6, 12]. This indicates early activation of resident immune cells such as microglia in the brain [12–14]. C-X-C Motif Chemokine Ligand (CXCL1) and NLR family pyrin domain-containing 3 (NLRP3) were upregulated in tissue and microglia from inflammation-sensitized brains, indicating that microglia are primed via the CXCL1/CXCR2 axis [15]. Moreover, microglia develop an early pro-inflammatory phenotype, which may explain the early cytokine storm after inflammation-sensitized HI [13]. This leads to the hypothesis that during infection the cytokine level is already high prior to the HI event that TH loses its protective capacity.

In the last years, the contribution of the infiltration of peripheral immune cells post-HI became of great interest for new studies [14, 16]. Under healthy conditions, neutrophils do not cross the blood-brain-barrier (BBB). In the case of neonatal HI, studies have shown an early infiltration of neutrophils in the brain parenchyma within the first 24 h post HI [17, 18]. Neutrophil activation in the brain can cause degranulation or the formation of neutrophil-extracellular-traps (NETs), with release of proteinases and reactive oxygen species (ROS) [14, 19]. This contributes to neuronal damage or BBB disruption [20]. The level of inflammation following HI may be influenced by the interaction between invading leukocytes and local immune cells such as microglia. Therefore, the investigation of immune cell signaling pathways may provide important information for the development of new therapeutic approaches.

To elucidate the pathological differences associated with inflammation-sensitized HI, the transcriptomic profiles of microglia from inflammation-sensitized rat brains following HI were investigated and compared with non-sensitized groups.

## Materials and methods

### Animals and experimental procedure

Parts of this study have been previously published by Serdar et al., 2020 [15]. All animal experiments were performed in accordance with the Animal Research: Reporting of in vivo Experiments (ARRIVE) guidelines with government approval by the State Agency for Nature, Environment and Consumer Protection North Rhine-Westphalia, Germany. Briefly, seven-days-old (P7) Wistar rat pups of both sexes were used in all experiments. All pups were kept at the central animal laboratory of the University Hospital Essen, Germany with a 12:12 h dark: light cycle at an environmental temperature of 21 °C with food and water *ad libitum*. Before the experiments, all animals were randomized across litters, sexes, and weights and all following experiments and analyses were performed by observers blinded to the different treatments [4, 6].

**Fig. 1:**
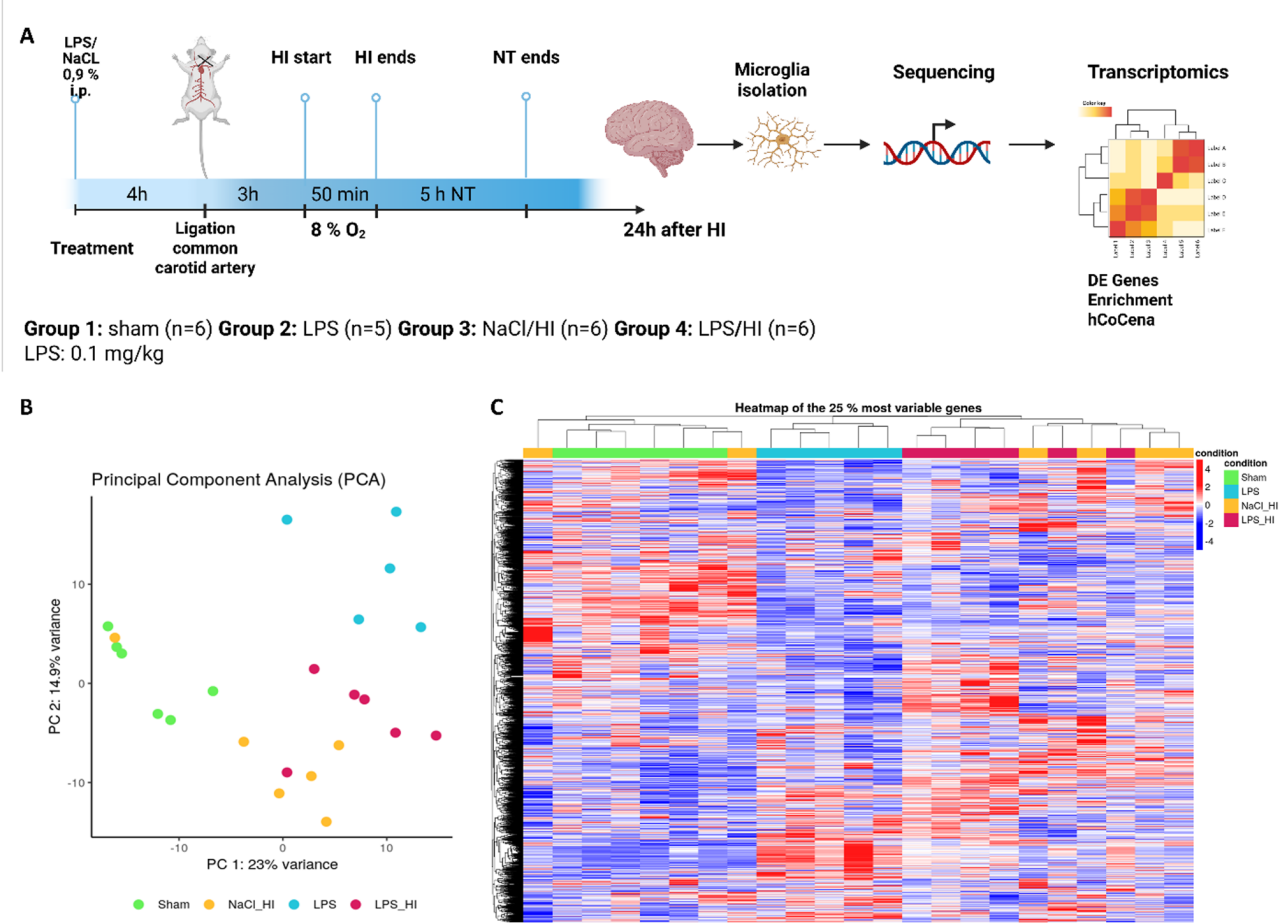
Transcriptomic analysis of microglia isolated from brains after different HI conditions. **A** Experimental design of the inflammation-sensitized neonatal HI model in 7-days-old Wistar rats, following isolation of microglia. Four hours before unilateral common carotid artery ligation group 2,3 and 4 received i.p. injection of LPS or NaCl. Group 3 and 4 underwent surgery followed by 50 min hypoxia (8% O_2_, 36 °C T_rectal_). After 5 h of normothermia (T_rectal_ 37 °C), the pups returned to their dams. 24 h after HI, microglia were isolated using CD11b-based magnetic cell sorting and RNA sequencing was conducted **B** PCA plot visualizes the relationship of all samples based on gene expression in isolated microglia, showing overlap of LPS/HI group with LPS and NaCl/HI group. **C** Heatmap of the 25% most variable genes in all four conditions. Sham and LPS group cluster together whereas the different samples of the NaCl/HI and LPS/HI groups are more spread in their gene expression

The temperature during handling and experimental procedures was monitored in sentinel pups, which were not further included in the different treatment groups. All the rat pups were kept on a servo-controlled mat (CritiCool, MTRE, Yavne, Israel) during separation from their dams. Their temperature was monitored by the sentinel pup via a rectal temperature probe (IT-21, Physitemp Instruments, Clifton, NJ, United States), continuously maintaining nesting temperature of P7 rats or treatment temperatures during experiments (see below). In the experimental setup, four groups were included:

(1) Sham group: underwent sham surgery (incision of the neck under isoflurane anesthesia (2% isoflurane) without further operation), (2) LPS group: received an intraperitoneal injection (i.p.) of lipopolysaccharide solution (LPS) (*Escherichia coli* lipopolysaccharide O55:B5, Sigma; 0.1 mg/kg) 4 h before operation and underwent a sham surgery, (3) NaCl/ HI group: received an injection of 0.9% NaCl, underwent a ligation of the left common carotid artery under isoflurane anesthesia and were afterwards exposed to hypoxia (8% O_2_) for 50 min at a rectal temperature (T_rectal_) of 36 °C, resulting in a mild HI injury as previously described [12] and (4) LPS/ HI group: received an i.p. injection of 0.1 mg/kg LPS 4 h before a left unilateral ligation of the common carotid artery was performed and were exposed to hypoxia as mentioned above (Fig. 1).

Immediately after the HI insult, the pups were kept at T_rectal_ of 37 °C for 5 h (normothermia treatment), as in our previous studies [4, 6]. After the treatment period, pups were immediately returned to their dam. At 24 h post-HI or sham period, all animals were sedated with chloralhydrate, and were decapitated. The brain tissue was removed and were used for different further analyses including western blot, qPCR and microglia isolation. A detailed description of total animal numbers used can be found in the original manuscript. In this manuscript we focused on isolated microglia cells.

### Magnetic activated cell sorting (MACS) of CD11 b/c positive microglia

A subset of animals was used for microglia isolation and further transcriptomic studies. Microglia were isolated 24 h after the operation by magnetic cell sorting based on CD11b/c positive marker. In groups (1) and (2), entire brains (including ipsi-/contralateral hemispheres) were used for analysis (*n* = 6 and 5 per group), whereas in groups (3) and (4), ipsilateral hemispheres were pooled to obtain a workable concentration of microglia (*n* = 12 animals with 2 hemispheres pooled per sample). For the mechanical and enzymatic dissection of the brains, the neural tissue dissociation kit by Miltenyi Biotech was used, followed by myelin removal according the manufacturer’s instructions (Miltenyi Biotech, Bergisch Gladbach, Germany). Afterwards the cell suspension was incubated with anti-CD11b/c coupled microbeads followed by magnetic separation of CD11b/c positive microglia.

### RNA sequencing and gene set analysis

RNA of the isolated microglia from the different treatment groups (see above) was isolated using Trizol (Thermo Scientific, Germany) and 500 ng of total RNA was processed using the TruSeq RNA Sample Preparation v2 Kit (low-throughput protocol; Illumina, San Diego, USA) to prepare the barcoded libraries. Libraries were validated and quantified using DNA 1000 and high-sensitivity chips on a Bioanalyzer (Agilent, Boeblingen, Germany); 7.5 pM denatured libraries were used as input into cBot (Illumina), followed by deep sequencing using HiSeq 2500 (Illumina) for 101 cycles, with an additional seven cycles for index reading. Fastq files were imported into Partek Flow (Partek Incorporated, Missouri, USA). Quality analysis and quality control were performed on all reads to assess read quality and to determine the amount of trimming required (both ends: 13 bases 5’ and 1 base 3’).

### Alignment of sequencing data

Quality control was performed using fastQC (v0.11.9)(https://github.com/s-andrews/FastQC?tab=readme-ov-file) and multiQC (v1.14) [21]. Sequenced reads were aligned against the rat reference genome mRatBN7.2 using kallisto (v0.48.0) [22] with an estimated average fragment length of 200 and an estimated fragment length standard deviation of 30. The alignment pipeline was executed using SnakeMake v7.20.0 [23].

### Data pre-processing

The following analysis steps were performed in R (v4.3.2) and RStudio (v2023.9.1.494). The count matrix was created by DESeq2 (v1.42.1) [24] using the tximport package (v1.30.0) [25]. Genes with less than 10 counts across all samples were excluded. Genes were annotated based on the mRatBN7.2 genome and gene symbols as well as biotypes were obtained from Ensembl [26] using biomaRt (v2.58.2) [27]. Non-coding genes with duplicated gene symbols were excluded and counts of protein-coding genes with duplicated gene symbols were summed and aggregated under one symbol. This resulted in 20,301 present genes for the analysis. Variance-stabilizing transformation and batch correction were performed with rlog (DESeq2) and limma/sva(v3.58.1/v3.50.0) [28]. Based on normalized counts, significant surrogate variables (SVs) were calculated using Permutation hypothesis testing according to the Buja and Exuboglu method with a *p*-value cutoff of 0.1. The three most significant SVs were considered for batch correction [29].

### Dimensionality reduction

A Principal Component Analysis (PCA) was conducted on batch-corrected and variance-stabilized gene expression counts using the stats package (v4.3.2). The first two principal components were visualized in a scatter plot generated with ggplot2 (v3.4.4).

### Differential gene expression analysis

Differential gene expression analysis based on DESeq2 was performed by adjusting *p*-values according to independent hypothesis weighting from IHW (v1.30.0) [30] at default settings and applying empirical Bayes shrinkage estimators from apeglm (v1.24.0) [31]. Genes with a fold change > 2 and an adjusted *p*-value < 0.05 were defined as differentially expressed genes (DEGs).

### Gene co-expression network analysis

Gene co-expression network analysis was performed using hcocena (v1.1.1) [32]. The 12,730 most variable genes of the normalized, were used as input, suggested by the *suggest_topvar* function followed by gene-gene correlations calculation using Pearson’s correlation. Gene pairs with a Pearson’s correlation coefficient lower than 0.832 were excluded from the network resulting in a network with 7,640 genes, 246,878 edges and an R²-value of 0.853. Leiden clustering with a resolution of 1.7 identified 18 modules with a minimum size of 15 containing a total of 7,252 genes. Hub genes of each cluster were determined using the package’s *find_hubs* function with default settings. The top 10 genes of each cluster were used for visualization.

### Functional gene enrichment analysis

To analyze selected sets of genes concerning their biological function, two different approaches were used: Gene Set Enrichment Analysis (GSEA [33], and Gene Set Variation Analysis (GSVA [34]). In both analyses, three publicly available databases were used for pathway analysis: Gene Ontology: Biological Process [35], Hallmark [36] and Reactome [37]. The gene sets related to the functional pathways were obtained from the Molecular Signature Database (MSigDB) from the files “m5.go.bp.v2023.1.Mm.symbols.gmt”, “mh.all.v2023.1.Mm.symbols.gmt” and “m2.cp.reactome.v2023.1.Mm.symbols.gmt”. For functional analysis of differentially expressed genes and genes within the clusters of the co-expression network, GSEA was performed using clusterProfiler (v4.10.0) [38]. All unique genes in the dataset were used as background genes for the enrichment. Multiple testing correction was performed using the Bonferroni method [39] with an adjusted *p*-value cutoff of 0.05. For testing the enrichment of otherwise specified gene sets, GSVA was performed. Gene-set-specific enrichment scores were calculated based on the normalized, batch-corrected counts using the GSVA package (v1.50.5) [34].

### Visualizations and statistics

Results were visualized using ggplot2 (v3.4.4) and related packages. Statistics were computed using the stats (v4.3.2) and the rstatix (v0.7.2) package. GSVA scores were calculated as outlined earlier and visualized as radar plots using ggradar (v0.2) (https://github.com/ricardo-bion/ggradar). Co-expression networks were visualized using Cytoscape (v3.10.3) [40]. Well-established marker genes from the literature were mapped onto the network using. Literature-known marker genes were visualized within the network using hcocena (v1.1.1). Normalized expression values of these markers were visualized as boxplots and compared using a Wilcoxon rank-sum test [41] with an Benjamini-Hochberg [42] adjusted *p*-value of 0.05 as cutoff. Heatmaps were constructed using pheatmap (v1.0.13) and ComplexHeatmap (v2.18.0) with euclidean distances as metric for clustering of rows and columns.

### Data availability

The RNA sequencing data generated and analyzed in this study have been deposited in the Gene Expression Omnibus (GEO) under accession number GSE294909.

## Results

### Transcriptomic analysis of microglia revealed clustering of differential expressed genes in different groups based on their conditions

To gain further insight into the processes of the immune system in the context of inflammation following HI, P7 Wistar rats were injected with LPS before left common-carotid artery ligation was conducted (Fig. 1). After 24 h, microglia were isolated from the brains and transcriptome analysis of sham, LPS, LPS/HI and NaCl/HI group was performed (Fig. 1A). Data quality was visualized with principal component analysis (PCA) showing clustering of groups depending on their gene expression. The PCA revealed clear clustering of the different groups and partial overlap observed between LPS/HI group, NaCl/HI and LPS group (Fig. 1B). In order to investigate this in detail, the 25% most variable genes were visualized in a heat map and samples were sorted by unbiased hierarchical clustering based on their transcriptomic profiles, which resulted in more than three clusters suggesting higher transcriptional heterogeneity. The sham and LPS group are separated in their clustering while the NaCl/HI and LPS/HI group are more spread between the clusters (Fig. 1C).

### Transcriptomic data revealed upregulation of genes involved in neutrophil chemotaxis

**Fig. 2:**
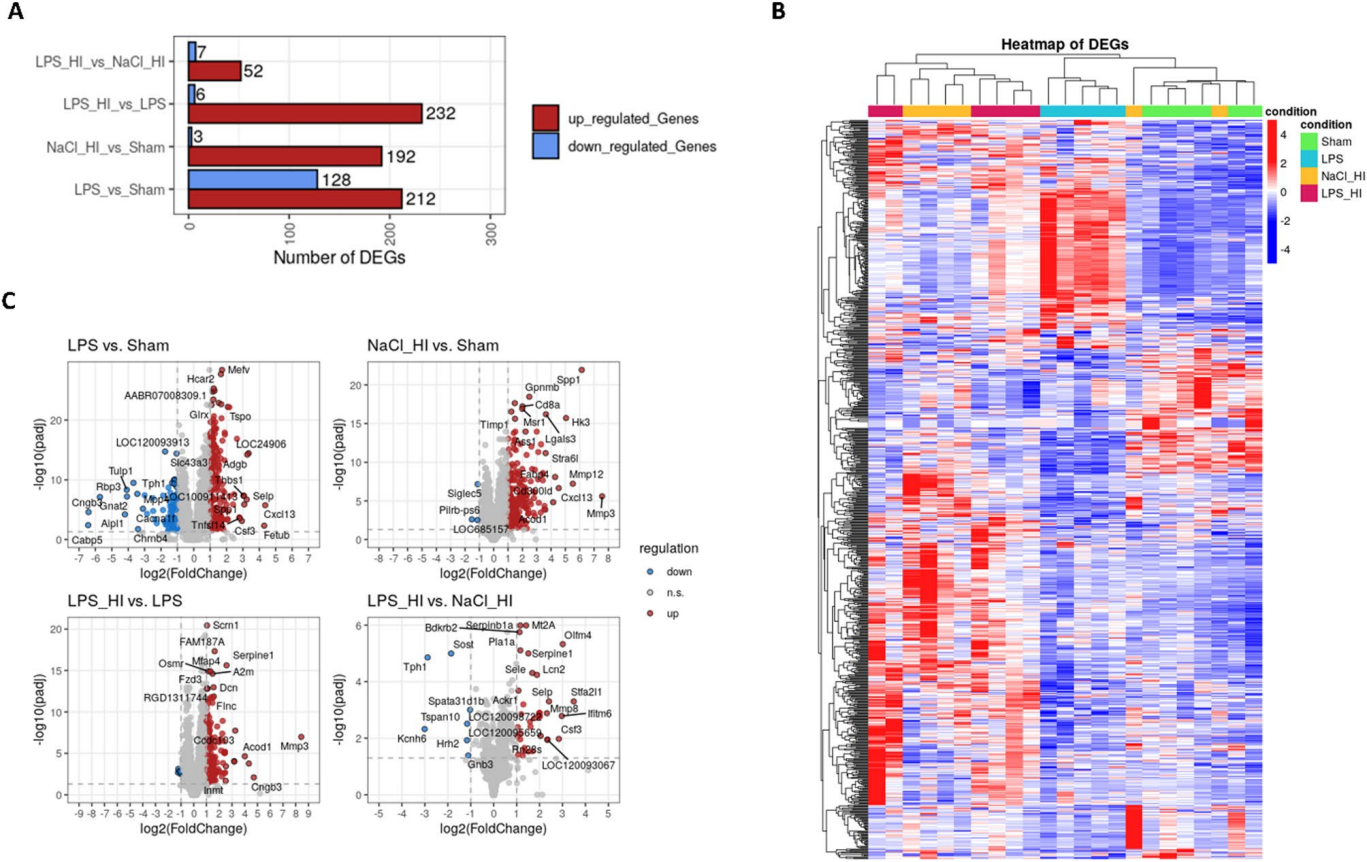
Transcriptomic analysis of isolated microglia 24 h after LPS/HI revealed differentially expressed genes in comparison to sham, LPS and NaCl/HI. **A** Number of differential expressed up- (red) and down (blue)- regulated genes between conditions. **B** Heat map showing differentially expressed genes in microglia isolated from the four treatment groups. The upregulated genes are shown in red and the down-regulated genes are shown in blue. **C** In the volcano plot log10 (*p*-value) was plotted against log2 (FoldChange) of gene expression. Significantly upregulated genes are indicated in red and downregulated genes are indicated in blue

**Fig. 3:**
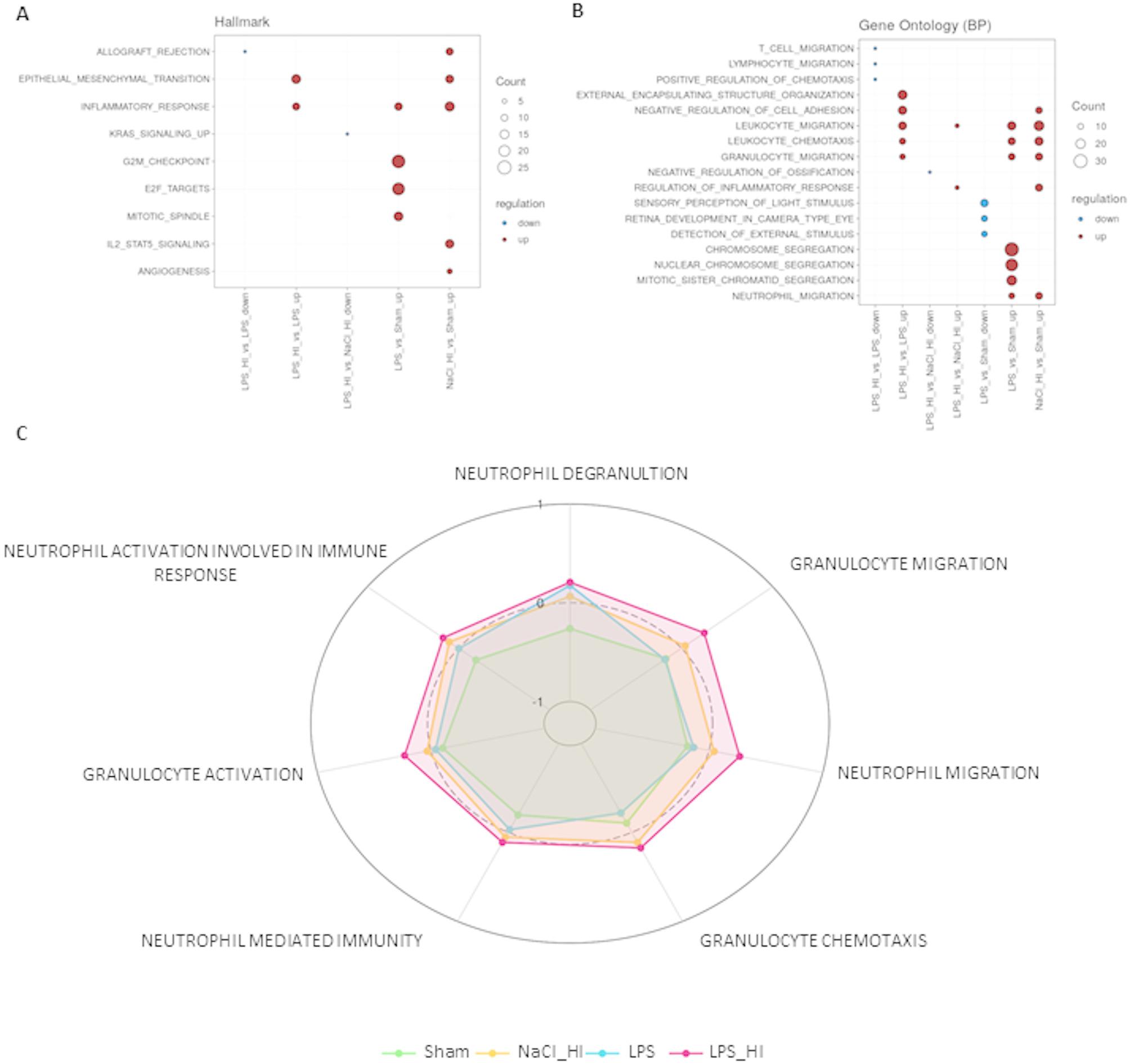
Functional Enrichment analysis. **A** Gene Set Enrichment Analysis based on Hallmark database revealed upregulation of inflammatory response in LPS/HI, LPS and NaCl/HI group. Upregulated counts are shown in red and downregulated counts are shown in blue. **B** Gene Set Enrichment Analysis based on gene ontology database revealed strong upregulation in leukocyte activation and migration pathways in LPS/HI group. Upregulated counts are shown in red and downregulated counts are shown in blue. **C** Gene Set variation Enrichment scores revealed the highest scores in neutrophil immune pathways in the LPS/HI group (red) compared to NaCl/HI (yellow), LPS (blue) and sham (green)

**Fig. 4:**
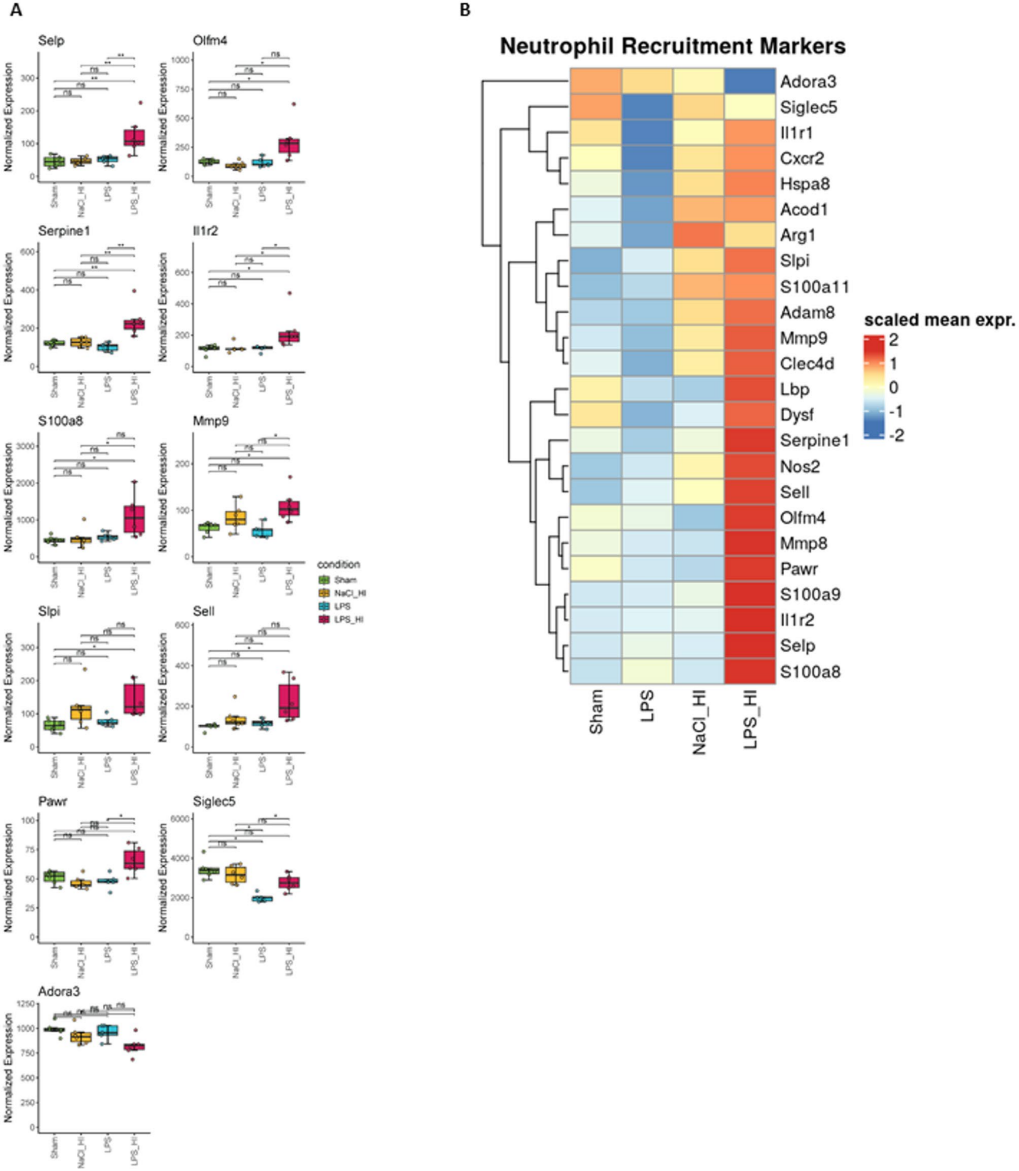
Normalized gene expression of markers involved in neutrophil recruitment and microglia priming. **A** Box Plot visualizes normalized gene expression of neutrophil recruitment markers of all treatment groups. The gene expression of Selp, Serpine1 and Il1r2 was significantly upregulated in the LPS/HI group compared to all other treatment groups (Wilcoxon rank-sum test, Benjamini-Hochberg adjusted *p*-value < 0.05). **B** Heat Map of neutrophil recruitment markers expression indicating high expression of those markers in the LPS/HI group

Based on the transcriptomic data, differential expressed genes (DEGs) in the four different treatment groups were compared (Fig. 2A, B), and the results visualized by volcano plots. As we were mainly interested in the differences in gene expression comparing LPS/HI to NaCl/HI or to LPS, we focused on these groups to find specific marker genes for inflammation-sensitized HI. The most upregulated genes in the volcano plot were e.g. Olfactomedin (Olfm4), Selectin P (Selp), Lipocalin 2 (Lcn2), Serpin family E member 1 (Serpine 1) and matrix-metallopeptidase 8 (MMP8), which contribute mostly to leukocyte chemotaxis and activation (Fig. 2C). This finding was also verified by the Gene Set Enrichment Analysis based on the Gene Ontology database, where granulocyte and neutrophil migration and chemotaxis had the highest counts in LPS/HI group compared to sham. Hallmark enrichment additionally revealed an upregulation of the inflammatory response in LPS/HI compared to LPS group (Fig. 3A). GSEA showed a clear upregulation of pathways contributing to neutrophil-mediated immunity, degranulation and migration in the LPS/HI group, supported by similar trends in GSVA with related terms (Fig. 3B, C). Based on these finding we checked our dataset for the regulation of genes mainly involved in inflammation response and leukocyte chemotaxis. We found several upregulated genes, which are contributing to neutrophil recruitment and activation (Fig. 4). Comparing LPS/HI to all other conditions Selp, Olfm4, Serpine1 and pro-apoptotic WT1 regulator (Pawr) were the genes which were significantly upregulated in all cases (Fig. 4A, B). This finding can indicate specific marker genes for an LPS/HI insult. The expression of S100a8 was not significantly different between the LPS/HI and LPS group but significant between LPS/HI and NaCl/HI conditions, indicating that this expression might be specifically LPS-triggered (Fig. 4A). Inflammation pre-sensitized HI-insult stimulated the expression of Matrix-Metallopeptidase (MMP9), Secretory Leukocyte Peptidase Inhibitor (Slpi) and Selectin L (Sell) when comparing expression level to the sham group (Fig. 4A).

### CoCena revealed clustering of leukocyte recruitment markers in two submodules

**Fig. 5:**
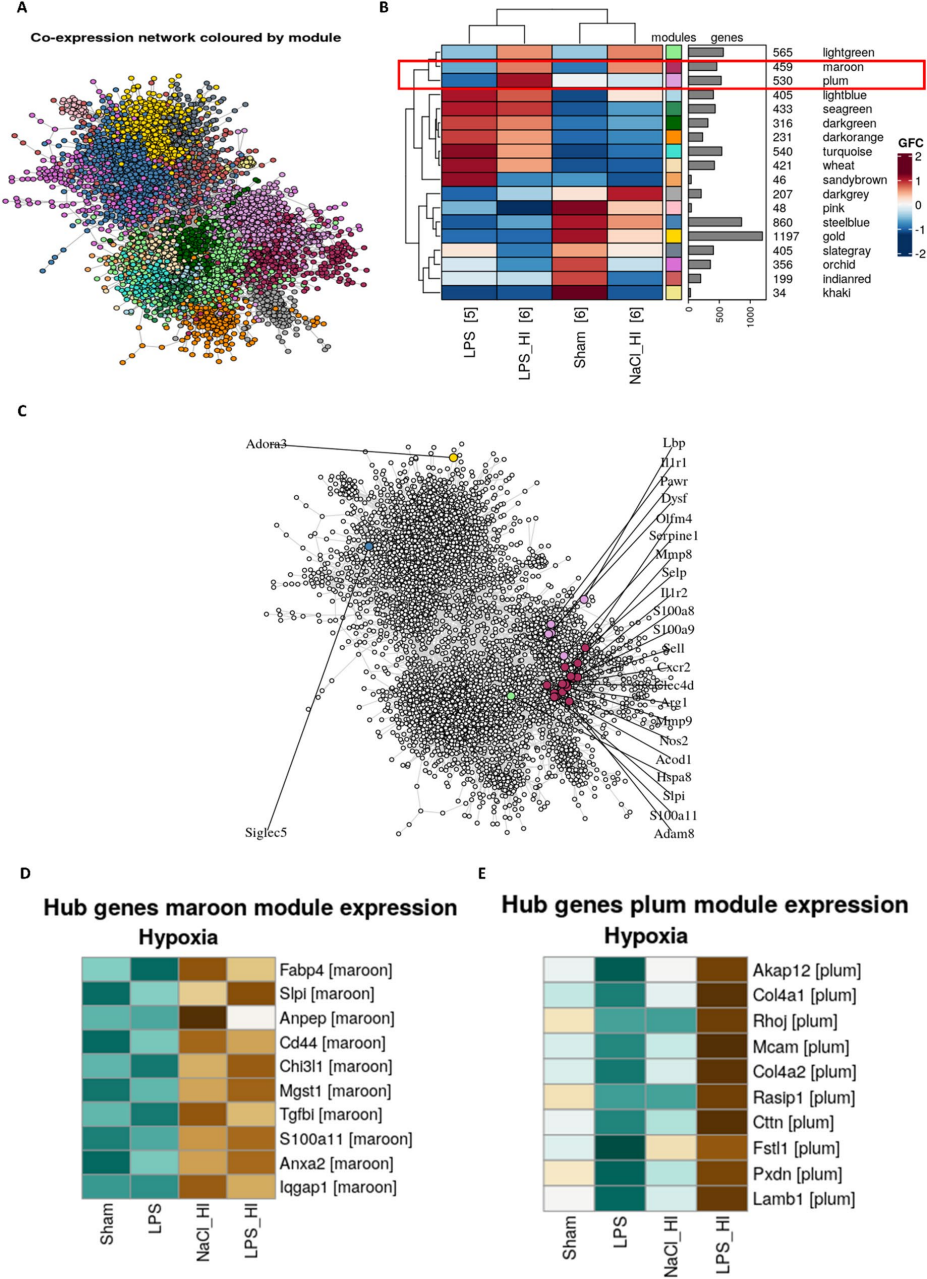
hCocena Network of gene-gene correlation. **A** Network analysis revealed 18 submodules based on similar gene expression. **B** Analysis of leukocyte recruitment marker in CoCena network revealed clustering in two groups (maroon and plum), which confirm similar expression patterns of leukocyte activation markers. Adora3 and Siglec 5 are the only genes that are not found in these clusters. **C** Heatmap of up- or downregulated genes in different groups based on CoCena network modules. **D** Hub-gene detection from the maroon hCoCena model identified regulating genes based on high connectivity. Expression of those genes in the different groups is visualized in a heatmap. **E** Hub-gene detection from the plum hCoCena model identified regulating genes based on high connectivity. Expression of those genes in the different groups is visualized in a heatmap

The transcriptomic data were further analyzed based on Construction of Co-expression network analysis (CoCena), where genes with similar expression pattern across all treatment groups were clustered in a co-expression network [43]. Leiden clustering of the network revealed 18 submodules (Fig. 5). Leukocyte recruitment genes clustered in two major modules (maroon and plum), both enriched in LPS/HI samples indicating a specific relevance of these genes in the LPS/HI condition. Sialic Acid Binding Ig-Like Lectin 5 (Siglec 5) and Adora3 did not cluster in the same module, indicating different expression patterns (Fig. 5). Overall, the co-expression network analysis revealed strong upregulation of leukocyte recruitment markers in the LPS/HI group compared to sham, LPS and NaCl/HI. Siglec5 and Adora3 were decreased in LPS/HI, as they play a role in anti-inflammatory pathways.

Hub-gene detection of the two CoCena modules (maroon and plum) revealed genes which are highly connected to other genes in this network and are likely to be involved in regulating transcription of those modules. The maroon cluster showed a stronger contribution to inflammation response and microglia activation as Slpi and S100a11 are found as hub-genes in this cluster (Fig. 5D). The hub-gene detection indicated that Slpi and S100a11 regulates gene expression of other genes involved in immune responses. GO-analysis and Reactome-analysis of this cluster could also confirm high counts for leukocyte migration (supplement, Fig. S1) and neutrophil degranulation (supplement, Fig. S2) The plum cluster showed hub-genes involved in metabolic, cell proliferation and migration processes like melanoma cell adhesion molecule (Mcam) [44], Rho GTPase j (Rhoj) [45] and Ras interacting protein (Rasip1) [46] (Fig. 5E). Hallmark enrichment analysis revealed strong clustering of hypoxia and angiogenesis marker genes in the plum cluster (supplement, Fig. S3).

## Discussion

Comparing expression levels of the four groups, Olfm4, Selp, Serpine 1, Il1r2 and Pawr were found to be the significantly upregulated genes in LPS/HI injury (Fig. 4). These findings can indicate that these genes are specific markers for inflammation-sensitized HI conditions in microglia. Olfm4 was significantly upregulated in the LPS/HI group compared to other groups. It is a glycoprotein known to be upregulated in inflamed tissue and to interact with MMP9 [47]. MMP9 expression was also upregulated in microglia from both HI groups and has been shown to act over Notch signaling pathway via p53 [47]. Olfm4 is expressed in high amounts in neutrophils and can serve as a biomarker for the severity of several bacterial or viral infections [48].

IL1R2 expression was upregulated after LPS/HI injury compared to other groups. High expression of IL1R2 is known to be induced by hypoxia and can activate the HIF1a pathway [49]. Activation of this receptor can promote angiogenesis and migration in response to HI insult. Interestingly, the receptor expression was not upregulated in the LPS or NaCl/HI group compared to sham, indicating the specific condition in the LPS/HI insult. In contrast to Il1R2 expression, Il1R1 expression was not significantly altered between microglia isolated from different groups (Fig. 4).

Serpine 1 was another gene which expression was significantly higher in the HI/LPS group compared to other groups (Fig. 4). Serpine 1 mainly acts as an inhibitor of tissue-type plasminogen activator and is involved in the degradation of blood clots. In adult stroke models, it was found to be important for neutrophil migration [50]. In previous studies, it was found to be a potential biomarker for acute cerebral infarction [51]. Our study could confirm high Serpine1 expression in microglia after inflammation sensitized HI, which may be another factor for a severe outcome after HI under inflammatory conditions.

In microglia from inflammation-sensitized brains isolated 24 h after HI, Selp-mRNA was highly expressed compared to other groups, leading to the hypothesis that in the case of inflammation during HI, Selp expression contributes to an over-activated immune system and strong activation of neutrophils and microglia. In stroke model it was already found that Selp contribute to alter BBB function and a more severe outcome after injury [52]. Selp is known to be involved in several immunological processes including platelet activation, microglia and neutrophil recruitment [53]. Selp is expressed on endothelial cells and activated platelets [53]. Recent studies about SELP-PSGL-1 axis in glioblastoma found high expression in Selp-mRNA in microglia and secretion of Selp [54]. Inhibition of Selp could ameliorate pathology [54]. This may suggest that Selp is involved in the regulation of resident microglia in human glioblastoma tumors. Sell as another selectin was also significantly upregulated in LPS/HI compared to sham (Fig. 4). It is expressed on microglia to bind to the L-Selectin ligand on epithelia cells [55]. The upregulation of selectins in LPS/HI showed a high trend to leukocyte recruitment, indicating that a severe outcome after inflammation-sensitized HI can contribute to an over activated innate immune system and a breakdown of the BBB.

The transcriptomic profile of microglia after LPS/HI suggests that microglia are primed to react via pro-inflammatory pathways and release chemokines to potentially attract neutrophils. The heterodimer complex composed of calcium-binding domains S100A8/A9 is upregulated in isolated microglia after LPS/HI. These proteins contribute to a microglial switch to a pro-inflammatory phenotype [56] and acts via the NFƙb-signaling pathway to promote apoptosis in oligodendrocyte progenitor cells [56]. It is known to be upregulated in serum of multiple sclerosis patients [56], in brains after sepsis [57] and is increased in Alzheimer’s disease [58]. S100A8/9 acts as damaged-associated-molecular pattern (DAMP) to attract neutrophils [57]. The transcriptomic data showed that the upregulation of S100A8/9 in microglia after LPS/HI contributes to the activation of a pro-inflammatory microglia phenotype and to the potential recruitment of neutrophils. S100a11 was found to be a hub-gene in the maroon model of the CoCena network regulating the immune response (Fig. 5D). This alarmin is known to act over TNFα signaling as a pro-inflammatory protein and might play an important role in immune response regulation after a LPS/HI insult [59]. Slpi expression was upregulated in activated microglia after LPS/HI and was confirmed to be a hub-gene in our model. Slpi can be secreted by microglia and astrocytes to inhibit proteases released by leukocytes and can inhibit inflammatory response via inhibiting pro-inflammatory cytokines [60]. In stroke model, it was found to inhibit brain damage [61]. Slpi contributes to tissue repair and homeostasis and seems to be a hub gene which plays a role in neuroprotective pathways. The plum CoCena network (Fig. 5E) revealed high expression of hub-genes involved in cell migration, and proliferation in the LPS/HI samples. Rhoj is known to regulate downstream proteins involved in proliferation and migration and can also contribute to apoptosis inhibition [45]. As microglia rely on fast migration, metabolomics and morphology changes after activation, the high expression of genes involved in these processes is indicated in the hub gene detection (Fig. 5E).

### Downregulation of genes involved in anti-inflammatory pathways after LPS/HI

In our data we observed a downregulation of Siglec 5- and Adora 3-transcripts in the LPS/HI group (Fig. 4). Siglec 5 as a member of the surface lectin family serves as a receptor mediating the inhibitory signaling of neutrophil and microglial activation [62, 63]. The Siglec receptors function as an immune checkpoint to balance immune response. Downregulation of Siglec 5 after LPS/HI can contribute to increased toxicity signaling of microglia and neutrophils [62]. Adora 3 contributes to the inhibition of neutrophil degranulation and activation of this adenosine receptor was shown to be neuroprotective [64, 65]. A recent study about inhibition of Adora 3 in microglia found enhanced phagocytosis and a reduction of white matter injury after chronic ischemia [66]. The downregulation of genes like Siglec 5 and Adora3 suggests that pathways which contribute to anti-inflammatory processes are mostly downregulated in microglia after LPS/HI compared to LPS group or NaCl/HI. These findings support the hypothesis that the immune response during LPS/HI undergoes an imbalance towards the pro-inflammatory phenotypes.

### Selectins and Serpine 1 were identified as promising markers for inflammation-presensitized HI

The transcriptomic profile of microglia from inflammation-sensitized HI brains not only showed upregulation of genes involved in neutrophil chemotaxis, activation and degranulation, it also showed downregulation of genes involved in inhibition of neutrophil activation. One of the major differences of the LPS/HI group compared to the other groups was the strong upregulation of the Selectin genes. The transcriptomic data revealed that microglia are primed to potentially attract innate immune cells like neutrophils to cross the BBB. Previous studies from our group showed an early infiltration of neutrophils within 24 h after HI [17]. The transcriptomic data from LPS-sensitized HI brains can support the theory that microglia contribute to neutrophil infiltration in the brain parenchyma early after HI. However, a unique and significant biomarker for the early stage of severe HI after inflammation was not yet found. Our data revealed that Selectin expression especially Selp showed promising results. In SARS-COV-19 studies, soluble Selp was identified as a diagnostic and prognostic biomarker [67, 68]. We could also confirm Serpine 1 expression to be a potential diagnostic marker for pre-sensitized HI as studies found Serpine 1 to be a serum marker for acute stroke [51].

There are some limitations in our study. First, we only focused on microglia activation 24 h after HI. As the developing brain quickly changes and adapts to new condition, other time points are necessary to elucidate the microglial profile over time. However, we previously showed a time dependent cytokine profile of isolated microglia from LPS/HI, where 24 h after insult the pro-inflammatory cytokine level was significantly increased [13].As we only focus on transcriptomic profiles, we cannot predict that the transcripts are fully translated. This can lead to another outcome at the proteome-level. To confirm the role of Selp in the LPS/HI model, a closer look at the protein level is necessary. Blocking Selp with a specific antagonist as outlined by Yeini et al., 2021 [54], could answer the questions whether this protein is necessary for immune cell infiltration and activation and whether its inhibition could lead to a positive outcome after HI.

Third, in this study, we did not include a hypothermia group, as our primary objective was to characterize the microglial state in the specific context of inflammation-sensitized HI injury. In future work, we plan to incorporate the LPS/HI/TH group to further investigate the mechanisms underlying the reduced efficacy of therapeutic hypothermia in this particular pathological setting. Fourth, the animal model which was used in the study is a mild HI model [69], as we include a LPS/HI cohort. Previous research has shown that the double-hit model, combining severe injury with LPS, leads to extensive brain damage and a high mortality rate [4, 6]. Since our aim is to process the brains for subsequent cell isolation, such severe injury would represent a significant limitation.

Last, in the present study, we did not examine sex-specific differences within the cohorts due to an insufficient sample size. It is known that transcriptomic profile could alter between female and male which we did not investigate in this study. Notably, our previous research found no significant sex-related differences in brain region atrophy or in the expression levels of CXCL1, CXCR2, and NLRP3.

We employed CD11b/c magnetic isolation beads for microglia isolation, a robust and widely used method. However, since macrophages also express these markers, contamination with infiltrating macrophages, particularly in the context of blood-brain barrier (BBB) disruption, cannot be entirely excluded. Furthermore, tissue dissection and isolation procedures are known to influence microglial activation states. To minimize such ex vivo activation, all samples were processed rapidly and under cold conditions.

## Conclusion

Our studies revealed the unique profile of microglia 24 h after LPS/HI showing a possible contribution to leukocyte and especially neutrophil recruitment and activation. Selp and Serpine 1 could be identified as promising markers for acute inflammation following HI. The fact that microglia express less anti-inflammatory markers can possibly indicate a strong over-activation of inflammatory pathways 24 h after LPS/HI in the brain compared to HI or LPS only. To this point microglia seemed to be the key players in acute brain damage, which we also could confirm in our studies. However, we also showed a relevant role of neutrophils in inflammation-sensitized brain injury indicating a possible interaction between microglia activation and neutrophil-mediated immune response. Targeting this cell-cell interaction might reveal new approaches for further treatment strategies.

## Electronic supplementary material

Below is the link to the electronic supplementary material.

Supplementary Material 1

## Data Availability

The RNA sequencing data generated and analyzed in this study have been deposited in the Gene Expression Omnibus (GEO) under accession number GSE294909.

## Abbreviations

Adora 3: Adenosine A3 receptor
BBB: Blood-brain-barrier
CXCL1 C-X-C: Motif Chemokine Ligand 1
CoCena: Construction of Co-expression network analysis
DAMP: Damage-associated-molecular pattern
DEG: Differential expressed genes
GO: Gene Ontology
HI: Hypoxic-ischemia
Il1r1: Interleukin1 receptor1
LCN2: Lipocalin 2
LPS: Lipopolysaccharide
MCAM: Melanoma cell adhesion molecule
MMP8/9: Matrix-Metallopeptidase 8
NLRP3: (NLR family pyrin domain containing 3)
Olfm4: Olfactomedin 4
Pawr: Pro-apoptotic WT1 regulator
Rasip1: Ras interacting protein1
Rhoj: Rho-related GTP-binding protein
ROS: Reactive oxygen species
Sell: Selectin L
Selp: Selectin P
Serpine 1: Serpin family E member 1
Siglec 5: Sialic Acid Binding Ig-Like Lectin 5
Slpi: Secretory Leukocyte Peptidase Inhibitor
S100A8/A9: S100 Calcium Binding Protein A8/A9
TH: Therapeutic hypothermia
