# Supplementary material for: Transcriptomic profile of microglia following inflammation-sensitized hypoxic-ischemic brain injury in neonatal rats suggests strong contribution to neutrophil chemotaxis and activation
Source: J Neuroinflammation. 2025 Jul 19;22:189. doi: 10.1186/s12974-025-03516-1 (PMC12276676; doi:10.1186/s12974-025-03516-1)
Supplement: Supplementary file 1 — Supplementary Material 1 [file 12974_2025_3516_MOESM1_ESM.docx]

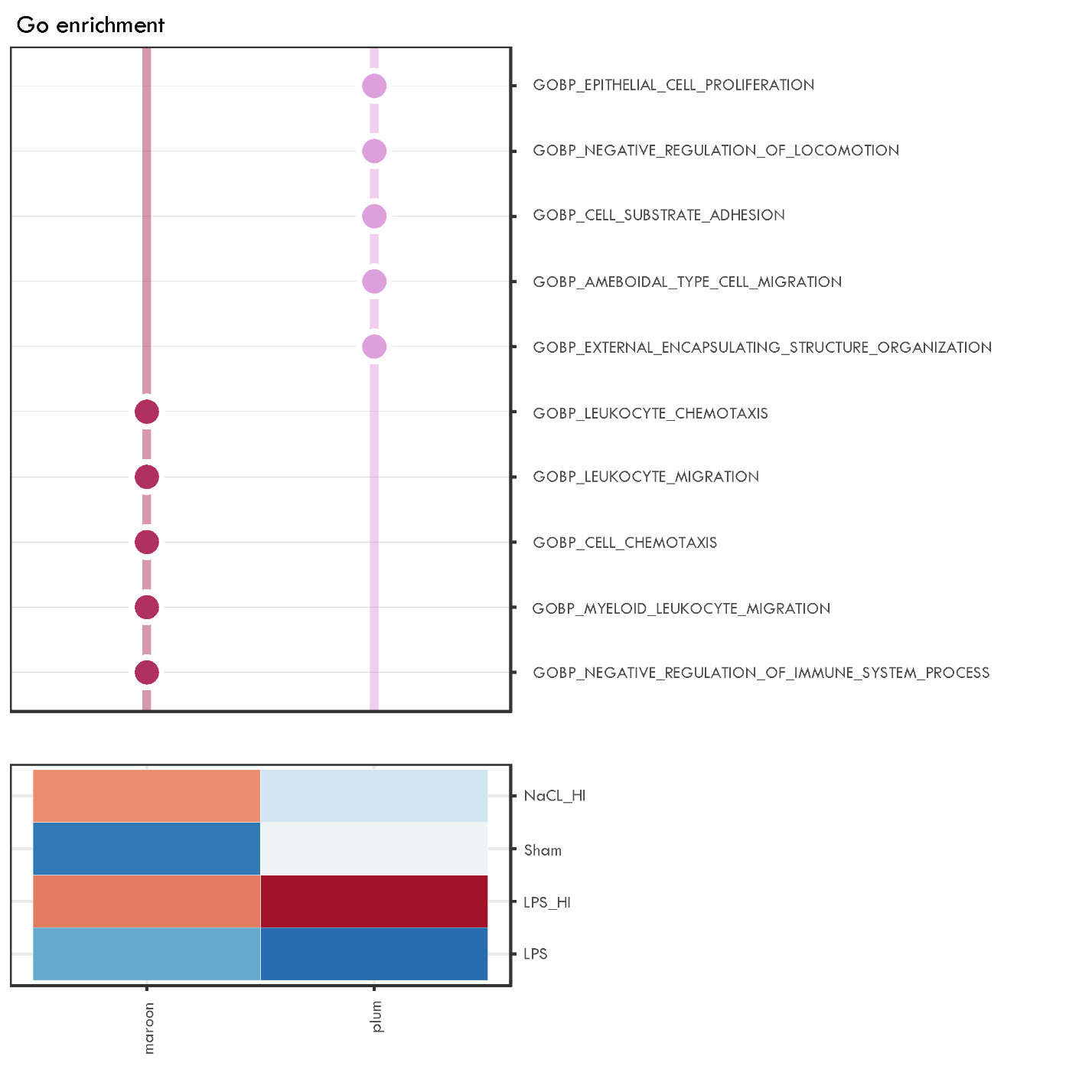


**Figure S1 Gene-Ontology Enrichment Analysis of the two hCocena clusters based on gene-gene correlation matrix.**


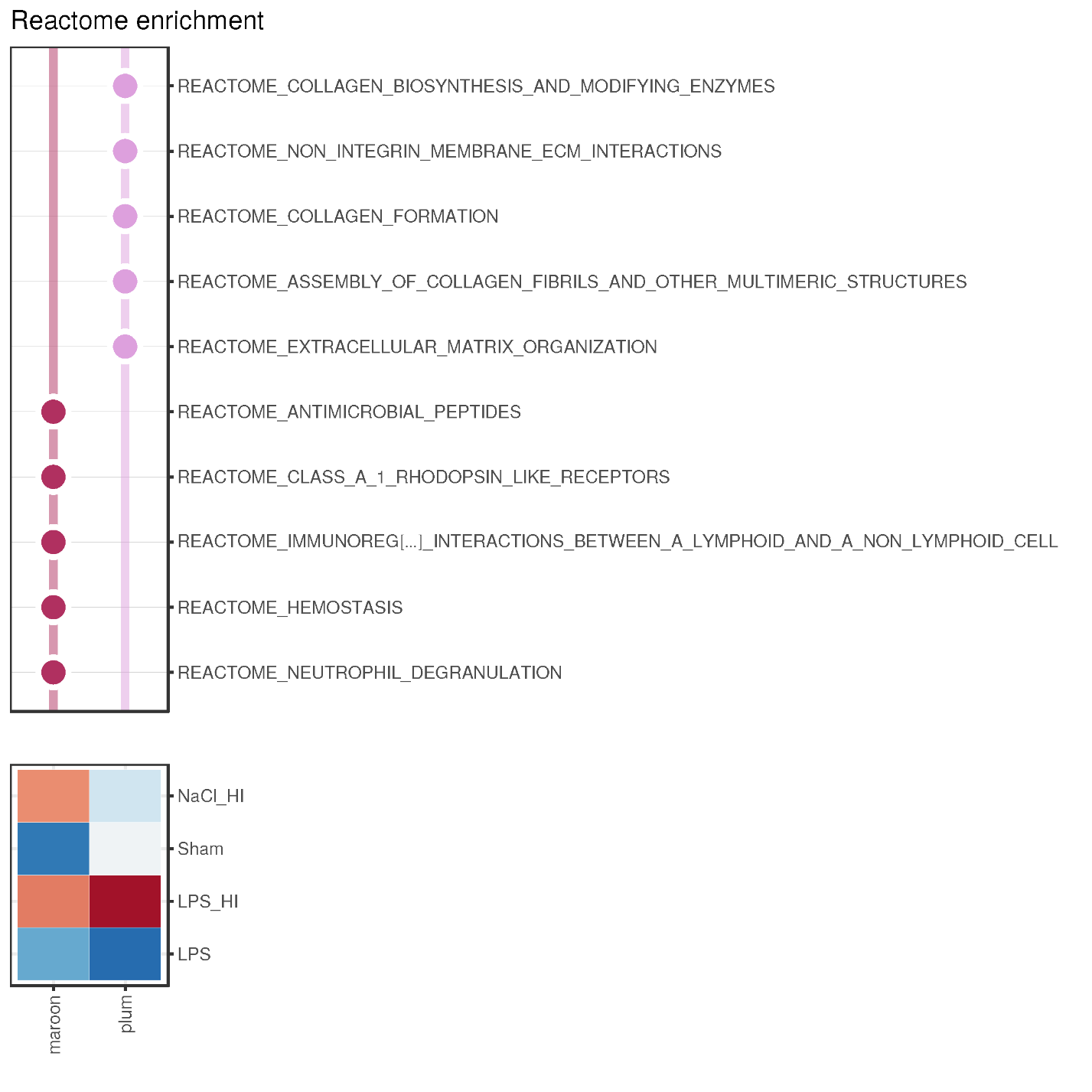


**Figure S2 Reactome Enrichment Analysis of the two hCocena clusters based on gene-gene correlation matrix.**


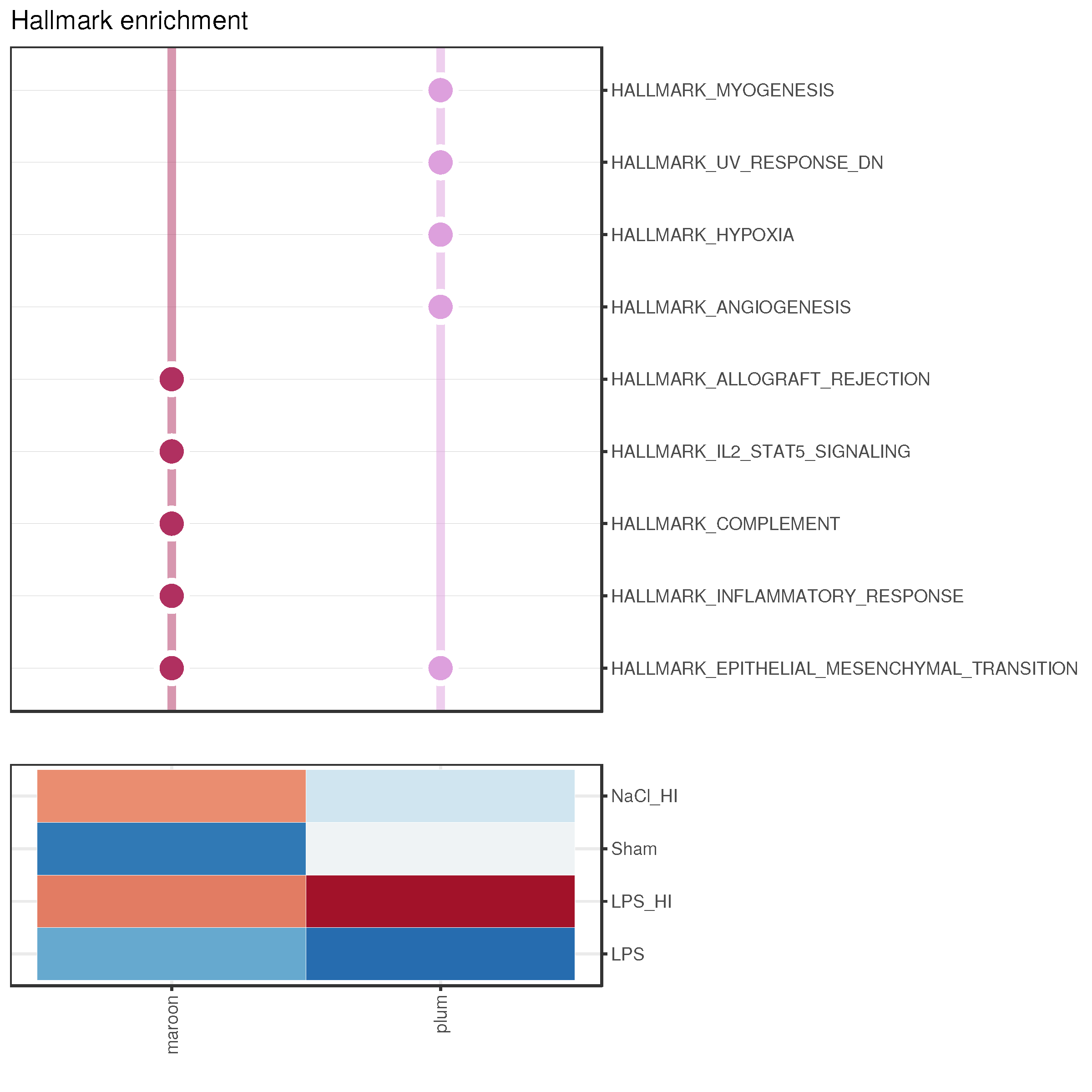


**Figure S3 Hallmark Enrichment Analysis of the two hCocena clusters based on gene-gene correlation matrix.**
